# Conservation of transcriptional elements in the obligate symbiont of the whitefly *Bemisia tabaci*

**DOI:** 10.7717/peerj.7477

**Published:** 2019-08-16

**Authors:** Dan-Tong Zhu, Chi Zou, Fei-Xue Ban, Hua-Ling Wang, Xiao-Wei Wang, Yin-Quan Liu

**Affiliations:** Institute of Insect Sciences, Zhejiang University, Hangzhou, China

**Keywords:** Portiera, Heat shock, Intergenic spacers, Transcriptional elements, *Bemisia tabaci*

## Abstract

**Background:**

Bacterial symbiosis is widespread in arthropods, especially in insects. Some of the symbionts undergo a long-term co-evolution with the host, resulting in massive genome decay. One particular consequence of genome decay is thought to be the elimination of transcriptional elements within both the coding region and intergenic sequences. In the whitefly *Bemisia tabaci* species complex, the obligate symbiont *Candidatus* Portiera aleyrodidarum is of vital importance in nutrient provision, and yet little is known about the regulatory capacities of it.

**Methods:**

*Portiera* genomes of two whitefly species in China were sequenced and assembled. Gene content of these two *Portiera* genomes was predicted, and then subjected to Kyoto Encyclopedia of Genes and Genomes pathway analysis. Together with two other *Portiera* genomes from whitefly species available previously, four *Portiera* genomes were utilized to investigate regulatory capacities of *Portiera*, focusing on transcriptional elements, including genes related with transcription and functional elements within the intergenic spacers.

**Results:**

Comparative analyses of the four *Portiera* genomes of whitefly *B. tabaci* indicate that the obligate symbionts *Portiera* is similar in different species of whiteflies, in terms of general genome features and possible functions in the biosynthesis of essential amino acids. The screening of transcriptional factors suggests compromised ability of *Portiera* to regulate the essential amino acid biosynthesis pathways. Meanwhile, thermal tolerance ability of *Portiera* is indicated with the detection of a σ^32^ factor, as well as two predicted σ^32^ binding sites. Within intergenic spacers, functional elements are predicted, including 37 Shine-Dalgarno sequences and 34 putative small RNAs.

## Introduction

Over the last two decades, multiple functions of microbiota have been investigated, from nutrition provision to reproduction (Stouthamer, Breeuwer & Hurst, 1999; Moran, McCutcheon & Nakabachi, 2008; Akman & Douglas, 2009). During the long-term associations, microbes have gone through genome decay and gene loss, resulting in tiny genomes (Moran, McCutcheon & Nakabachi, 2008). On one hand, protein-coding regions of these tiny genomes, which are easily accessible, have received broad attention. Considerable efforts have been made to elucidate the mechanisms underpinning the symbiosis by investigating protein-coding regions, and have yielded insights into the dynamics and functions of symbionts within the host (Zientz, Dandekar & Gross, 2004). On the other hand, intergenic spacers (IGS) between protein-coding regions comprising of non-coding regions receive little attention. This situation has improved recently due to high-throughput sequencing method, which has allowed us to identify functional elements involved in gene regulation within IGSs (Degnan, Ochman & Moran, 2011; Hansen & Degnan, 2014). Questions concerning why microbiota choose to retain some regulatory elements in IGSs (Moran, Dunbar & Wilcox, 2005), are of particular interest.

The whitefly *Bemisia tabaci* is a genetically diverse group including multiple morphologically indistinguishable cryptic species (De Barro et al., 2011). Among this species complex, two invasive species, Middle East-Asia Minor 1 (MEAM1, also known as B whitefly) and Mediterranean (MED, also known as Q whitefly), can cause severe damages to crops and plants around the world (Liu et al., 2007). There are also indigenous whitefly species, for instance, Asia II 3 and China 1 in China, causing less harm (Hu et al., 2011). The symbionts in whitefly are of vital importance, contributing to nutrient provision (Calle-Espinosa et al., 2016; Santos-Garcia et al., 2018) and host fitness (Shan et al., 2017; Lv et al., 2018). *Portiera*, as the obligate symbiont, is uniformly harbored by all species of whiteflies (Thao & Baumann, 2004). Studies on *Portiera* from the two invasive species of whiteflies MEAM1 and MED, have indicated that this bacterium mainly retains essential amino acid (EAA) biosynthesis genes (Santos-Garcia et al., 2012; Sloan & Moran, 2012; Jiang et al., 2013). Besides, sequenced *Portiera* genomes contain around 30% IGSs, which is much higher than that of other obligate endosymbionts from insects (Shigenobu et al., 2000; Nakabachi et al., 2006). With such a largely reduced gene set and a high proportion of IGSs, compelling questions focusing on the regulatory capacities of *Portiera* arise. However, research on this issue has been restrained by various factors such as: (i) IGSs contain functional elements such as sRNAs and nonfunctional regions like decaying pseudogenes fragment, which limits their recognizability; (ii) *Portiera* in bacteriocytes is clustered with other secondary symbionts (Rao et al., 2015) and is hard to be isolated from insect tissues; (iii) *Portiera* cannot be cultured *in vitro*.

In this study, in order to obtain information about overall regulatory capacities in *Portiera* genomes, we first sequenced the *Portiera* genomes from two indigenous whitefly species (Asia II 3 and China 1). We then analyzed the gene sets of these two *Portiera* genomes, together with two other *Portiera* genomes derived previously from two other species of whiteflies, MEAM1 and MED, of the *B. tabaci* complex. By comparing four *Portiera* genomes, we: (i) screened the transcription-related genes by using OrthoMCL and BLASTP; (ii) demonstrated two σ^32^ binding sites experimentally; (iii) identified the functional elements located in IGSs using a phylogenetic foot-printing approach.

## Materials & Methods

### Genome resources

Two *Portiera* genomes from two indigenous whitefly species (*B. tabaci* Asia II 3 and China 1) were sequenced and assembled according to a previously published protocol (Zhu et al., 2017). Genome sequences of *Candidatus* Portiera aleyrodidarum from *B. tabaci* Asia II 3 and China 1 were submitted to the GenBank databases under accession numbers CP016327 and CP016343. Another three *Portiera* genome sequences (*Candidatus* Portiera aleyrodidarum BT-B, *Candidatus* Portiera aleyrodidarum BT-Q and *Candidatus* Portiera aleyrodidarum TV) were downloaded from National Center Biotechnology Information (NCBI), and the accession numbers were listed in Table 1. In total, five *Portiera* genome sequences were analyzed and compared, with *Portiera*-B abbreviated for *Candidatus* Portiera aleyrodidarum from *B. tabaci* MEAM1 species, *Portiera*-Q for *Candidatus* Portiera aleyrodidarum of *B. tabaci* MED, *Portiera*-Z1 for *Candidatus* Portiera aleyrodidarum of *B. tabaci* Asia II 3, *Portiera*-Z3 for *Candidatus* Portiera aleyrodidarum of *B. tabaci* China 1 and *Portiera*-TV for *Candidatus* Portiera aleyrodidarum of greenhouse whitefly *Trialeurodes vaporariorum*.

**Table 1:** General feature of five *Portiera* genomes.

	***Portiera*-Z1**	***Portiera*-Z3**	***Portiera*-B**	***Portiera*-Q**	***Porteira*-TV**
Genome size, bp	354,523	359,359	358,242	357,472	280,663
CDS number	250	247	256	255	267
Intergenic spacers, %	32.9	39.4	33.5	33.5	7.7
GC content, % (Protein-coding region)	26.7	26.4	26.7	26.7	24.4
GC content, % (Intergenic spacers)	24.9	25.6	25.1	24.9	28.4
Accession number	CP016327	CP016343	CP003708	CP003835	CP004358

### Phylogenetic analysis

Whitefly species phylogeny was determined first by using whitefly mitochondrial cytochrome oxidase I (COI) genes downloaded from NCBI database. The tree was built using MrBayes (v.3.4.2) (Ronquist & Huelsenbeck, 2003), with the best-fitting model (TIM3 + G) generated by jModelTest (Darriba et al., 2012). For *Portiera* phylogenetic analysis, *Candidatus* Portiera aleyrodidarum TV served as an out-group member according its host taxonomic system. To obtain the homologous genes among the five *Portiera* genomes, protein-coding regions were extracted from each genome and subjected to OrthoMCL (Li, Stoeckert & Roos, 2003) with default parameters for orthologs identification. A total of 218 genes were defined as core-genes among the five genomes. The protein sequences of core-genes were concatenated, and aligned using MUSCLE (Edgar, 2004). The phylogeny was conducted by using Mrbayes, with the best-fitting model (cpREV + G + F) determined by ProtTest (Abascal, Zardoya & Posada, 2005).

### Recognization of transcription-related genes

To focus on the *Portiera* from the whitefly *B. tabaci* only, 227 core-genes were determined by OrthoMCL among the four *Portiera* genomes from whitefly *B. tabaci*, and subjected to BLASTP (*E*-value <10^−5^) and the Kyoto Encyclopedia of Genes and Genome (KEGG) Automatic Annotation Server tool (Moriya et al., 2007) for functional prediction. *Escherichia coli* K12 (commonly used as a model organism) (Keseler et al., 2011) served as a reference to predict transcription-related genes (mainly the amino acid biosynthetic pathways) and heat regulon within the protein-coding genes of *Portiera* genomes. *Ka/Ks* ratios between different gene pairs of *cspA* were calculated using DnaSP (v 5.10.1) (Librado & Rozas, 2009). σ^32^ binding sites in the regulatory region of genes were determined based on consensus sequences of σ^32^ binding sites in *E. coli* (CTTGAAA [11–15] CCCCATnT) with the criteria previously described (Wilcox et al., 2003).

### MED whitefly heat shock treatment

Insects are ectotherms and they are not able to control body temperatures. Hence, the elevation of environmental temperature will influence the bacterial symbionts within whitefly. In order to investigate possible function of predicted σ^32^ binding sites, MED whiteflies were subjected to a high temperature of 40 °C for 3 h, 25 °C as control. This high temperature of 40 °C has been shown to cause thermal stress of whiteflies (Wang et al., 2019). Temperature exposure experiments were conducted in climatic chambers (Sanyo, MLR-350H; Sanyo Electric Co., Ltd., Osaka, Japan) which offered precise control of temperatures within ± 0.5 °C of the set value. Newly emerged whiteflies were placed in tubes (2.5 cm × 0.5 cm diam) covered with gauze, and then exposed to 40 °C and 25 °C (control) for 3 h.

DNA (lysis buffer method) (Frohlich et al., 1999) and RNA (TRIzol protocol) (Rio et al., 2010) were extracted from whitefly samples, four biological replicates for DNA and 4 for RNA, with each replicate containing five males and five females. RNA was then reverse transcribed into cDNA using PrimeScript RT reagent Kit (Takara, Japan) after RNase-free DNase I treatment. Both DNA and cDNA samples were then subjected to quantitative PCR (qPCR) according to manufacturer’s instructions. The primers utilized were listed in Table S1. The qPCR results were calculated using the comparative *C*_*T*_ method (2^−ΔΔ*CT*^). The 2^−ΔΔ*CT*^ method normalized the amount of target genes in each sample against whitefly nuclear gene encoding beta-Actin (DNA samples), and gene expression level of target genes against whitefly nuclear gene encoding the ribosomal protein L29 (RPL29) (cDNA samples) (Li et al., 2013). The significance level was determined by using Student’s *t*-test.

### Functional elements searching in intergenic spacers

Orthologous IGSs were defined as sequences flanked by orthologous genes in genomes when comparing different genomes (Degnan, Ochman & Moran, 2011). IGSs from four *Portiera* genomes (from whitefly *B. tabaci*) were extracted and clustered in groups based on orthologous relationship. The lengths of paired orthologous IGSs were scattered to investigate the length variations between *Portiera* genomes from different species to examine their relationship. The orthologous IGSs were then aligned with MUSCLE. Identically aligned loci with more than 20 nucleotides in length were extracted and subjected to further analysis using a variety of means to investigate functional elements retained. Shine-Dalgarno sequences were predicted by RBSfinder (Suzek et al., 2001), with default parameters. Putative small RNAs were selected by using RNAFold (Lorenz et al., 2011), only if the minimum free energy was below 0 kcal/mole.

These transcriptional elements were investigated in another three *Portiera* genomes available, *Portiera*-TV and two other *Portiera* genomes (from *Aleurodicus dispersus* and *Aleurodicus floccissimus*, NCBI accession number LN649255 and LN734649). According to the orthologous IGSs containing the transcriptional elements in the *Portiera* genomes from *Bemisia tabaci*, sequences of the orthologous IGSs in the other three *Portiera* genomes (not from whitefly *Bemisia tabaci*) were extracted, and only sequences with a length over 20 nucleotides were kept. The investigation of transcriptional elements was performed as described previously.

## Results and Discussion

The genome of *Candidatus* Portiera aleyrodidarum BT-Z1 from whitefly Asia II 3 was 354,523 base pairs (bp) and contained 288 protein coding genes. For *Candidatus* Portiera aleyrodidarum BT-Z3 from whitefly China 1, the genome size was 359,359 bp, with 291 protein-coding genes identified. Both *Portiera* genomes were AT-rich, with 33 tRNA-coding genes and three rRNA-coding genes (5S, 16S and 23S). Together with *Portiera*-B from the whitefly MEAM1 and *Portiera*-Q from the whitefly MED, *Portiera* genomes in these four species of whiteflies (*Bemisia tabaci*) showed higher (than *Portiera* genome from whitefly *Trialeurodes vaporariorum*) similarity in genome size, coding sequence number and GC content (Table 1).

### The divergence and function of Portiera

**Figure 1:**
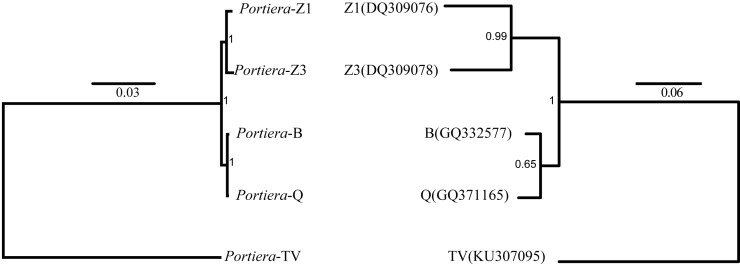
Bayes trees showing the phylogeny of *Portiera* and whitefly gene *mitochondrial cytochrome oxidase I* (*COI*) from five whitefly species. *Portiera* analysis was based on 218 core-genes determined by OrthoMCL. *Portiera* genome and *COI* gene from *Trialeurodes vaporariorum* served as out-group members, respectively. Posterior probabilities are given on the nodes.

In order to determine the phylogeny of *Portiera* in whitefly species, 218 orthologs among five *Portiera* genomes were generated, including four *Portiera* genomes from *B. tabaci* whitefly species and the *Portiera* genome from the greenhouse whitefly *T. vaporariorum* (as an out-group member). Sequence similarities between different *Portiera* pairs were calculated (Table S2). Interestingly, *Portiera*-B and -Q from invasive whitefly species showed the highest similarity (99.8%), and meanwhile, *Portiera*-Z1 and Z3 also present strong similarity (99.5%). However, the similarity of *Portiera* genomes between *B. tabaci* whitefly and greenhouse whitefly was only about 77%. The classification of *Portiera* was also evidenced by the phylogenetic tree, where four *Portiera* genomes from *B. tabaci* obviously formed two different clades, in coincidence with the classification of whitefly *B. tabaci* species (Fig. 1). Previous study on the divergence of whiteflies has implied that the divergence of the greenhouse whitefly *T. vaporariorum* and whitefly *B. tabaci* is dated back to 85.95 Ma, while the divergence of the species of whitefly *B. tabaci* is quite recent, approximately 17.73 Ma (Santos-Garcia et al., 2015). We believe that long-term co-existence within respective whitefly species following the divergence of whiteflies, probably contributes to the differences of *Portiera* genomes. Meanwhile, it is also revealed that although *B. tabaci* lineage (*Portiera* genomes from whitefly *B. tabaci*) is evolving faster than other lineages, the diversity among the *B. tabaci* lineage is smaller when compared to other lineages (Santos-Garcia et al., 2015). Our results on the similarity (Table S2) and phylogeny (Fig. 1) are in accordance with this finding.

**Figure 2:**
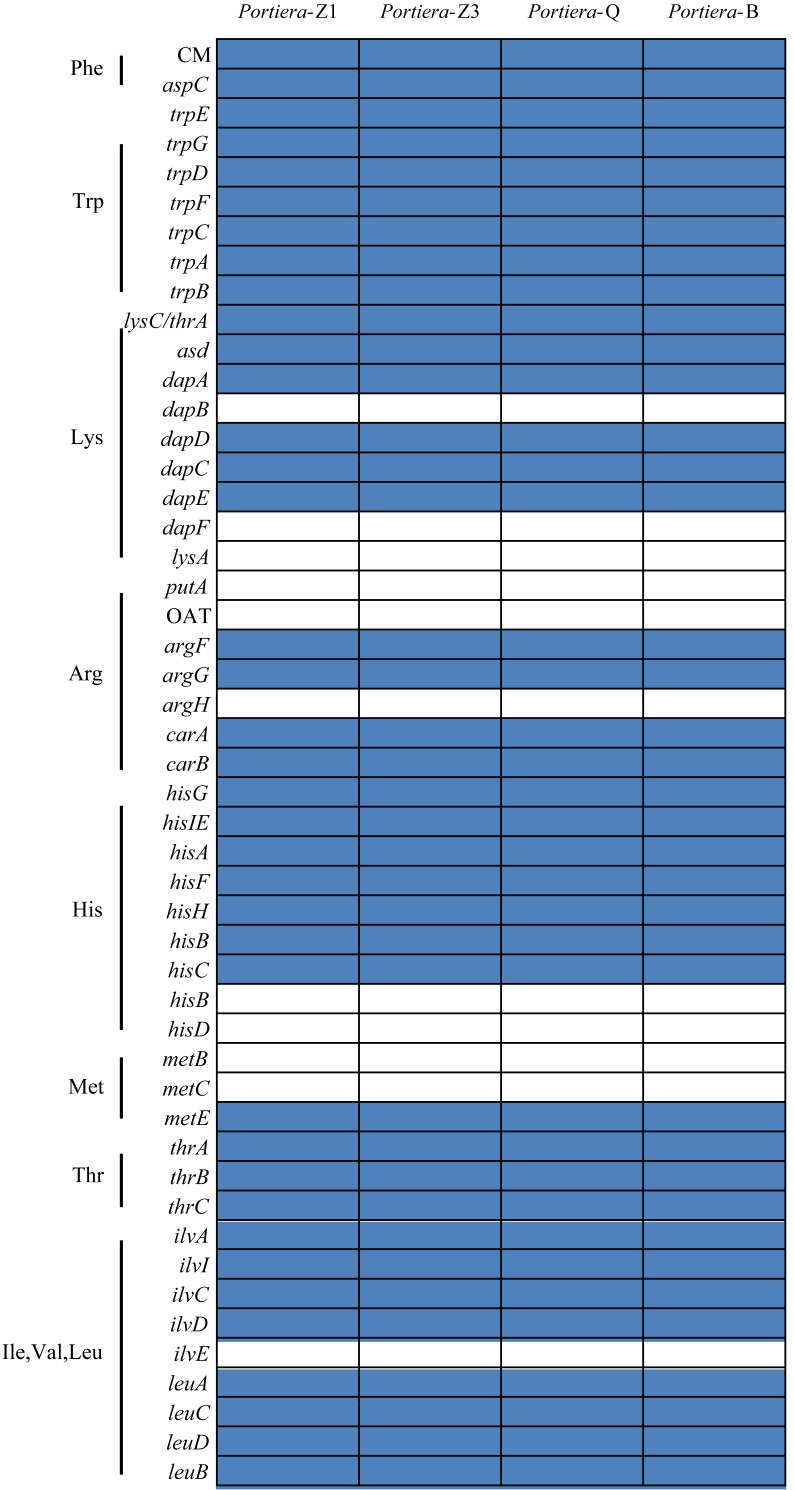
Genes involved in amino acids biosynthesis pathways present in four *Portiera* genomes. The blue rectangle indicates that the gene is present in the genome.

Despite the differences, from the orthologs and KEGG pathway analysis, two *Portiera* genomes from indigenous whitefly species in this study kept an identical set of genes dedicated to EAA biosynthesis and metabolisms (Fig. 2), indicating that *Portiera* in indigenous whitefly species may also be responsible for EAA provision. Meanwhile, *Portiera*-TV and two other *Portiera* genomes (from *Aleurodicus dispersus* and *Aleurodicus floccissimus*, NCBI accession numbers LN649255 and LN734649) are all predicted to contain the ability in amino acid biosynthesis (Santos-Garcia et al., 2015). Our results provided further evidence that *Portiera* enters into whitefly before the divergence, and is conserved as an essential part of whitefly genome, contributing to EAA provision.

### Genes related with transcription

To generate and compare a putative set of genes globally retained by *Portiera* from *B. tabaci*, 227 orthologous genes were determined from four *Portiera* genomes of whitefly *B. tabaci*. Regulatory genes related with amino acid biosynthetic pathways were all missing compared to *E. coli* (Table S3). The only transcription factor found in *Portiera* was gene *cspA*, which encodes a major cold-shock protein in *E. coli* (Schindelin et al., 1994). Besides, purifying selection was found acting on this gene (Table S4).

The lack of EAA regulation genes means a crippled ability of *Portiera* to regulate the biosynthesis of EAA. On the other hand, it has been pointed out that the genome of whitefly contains several genes that are within EAAs biosynthetic pathways (Luan et al., 2015). Hence, those genes from whitefly genome, other than genes from *Portiera* genomes, are supposed to be possible candidates that regulate biosynthesis pathways of EAAs.

In addition, some other common regulatory elements were detected. Three genes (*nusA, nusG* and *rho*) involved in *transcriptional termination were present, indicating that the* termination of transcription in *Portiera* may fall into both intrinsic termination and *rho*-dependent termination (Nudler & Gottesman, 2002). Core RNA polymerase (α_2_ββ′) was supposed to be complete, with the presence of *rpoA*, *rpoB*, and *rpoC*, which encodes α, β, and β′ subunit. RNA polymerase (RNAP) is of transcriptional importance in bacteria. Two σ factors functioning in the RNAP recognition of promoter sequences, were also suggested by the *Portiera* genomes. Along with primary σ factor σ^70^ (encoded by *rpoD*), σ^32^ (encoded by *rpoH*) was also detected, which is similar to *E. coli* (Helmann & Chamberlin, 1988) and *Buchnera* in aphids (Wilcox et al., 2003). Generally, this σ^32^ factor can compete with σ^70^, when the bacterium is confronted with heat stress, to redirect RNAP to promoters which contain σ^32^ binding sites.

### Heat shock treatment

**Figure 3:**
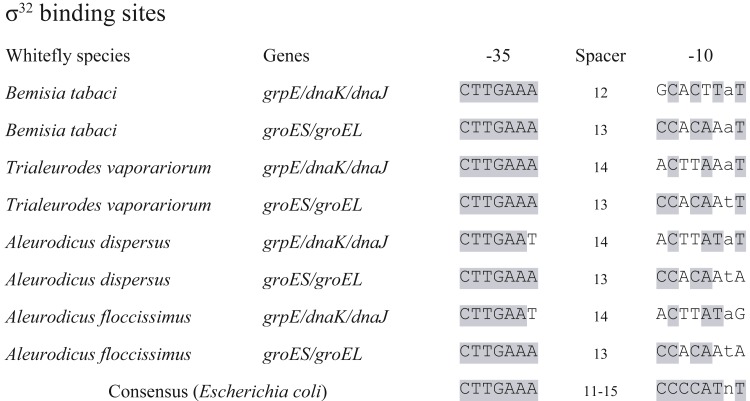
*σ*^32^ binding sites conserved in seven *Portiera* genomes. Whitefly species indicates the origin of the *Portiera*. ‘*Bemisia tabaci*’ contains four species of whitefly *Bemisia tabaci*. The *σ*^32^ binding sites were determined based on the consensus sequence of that in *Escherichia coli* with certain criteria. The grey shaded loci mean the same nucleotides as that in consensus sequence.

The detection of σ^32^ factor led to a set of 14 genes retained in *Portiera* as heat shock regulon, according to heat shock regulon of *E. coli* (Nonaka et al., 2006) (Table 2). These genes are predicted to encode relevant proteins responsible for cellular homeostasis, which indicates that heat shock response is probably partially conserved in *Portiera.* Meanwhile, promoter loci of the 14 genes were screened, with two possible σ^32^ binding sites detected (Fig. 3). Intriguingly, the σ^32^ binding sites were conserved among 4 *Portiera* genomes in whitefly *B. tabaci*, with identical −35 and −10 loci. Additionally, in *E. coli* and *Buchnera* genomes from aphids, σ^32^ binding sites are also found before genes *grpE* and *dnaKJ* (respectively), suggesting a possible universal mechanism of conserving σ^32^ recognition sequences before these three genes (Wilcox et al., 2003).

**Table 2:** Expression level variation of heat shock regulon from *Portiera*. The dynamics (FoldChange, FC) were determined by quantitative real-time PCR analysis of gene expression under heat shock (40 °C) and normal temperature (25 °C). Significance level was determined by using *t*-test.

Genes	Log2FC	FC (MEAN ± S.E.M.)	*p*-value
*groES*^b^	2.96	7.79 ± 1.949	^*^
*dnaK*^a^	2.66	6.31 ± 0.868	^***^
*ftsH*	2.64	6.24 ± 0.809	^***^
*dnaJ*^a^	2.42	5.34 ± 1.174	^*^
*grpE*^a^	2.26	4.79 ± 1.606	^*^
*hslV*	2.04	4.12 ± 0.642	^**^
*lon*	1.74	3.33 ± 0.523	^**^
*ybeY*	1.67	3.18 ± 0.773	^*^
*groEL*^b^	1.65	3.14 ± 0.347	^**^
*ileS*	1.55	2.92 ± 0.383	^**^
*rpoH* (*σ*^32^ factor)	1.54	2.91 ± 0.325	^**^
*valS*	1.46	2.75 ± 0.237	^***^
*hslU*	1.39	2.61 ± 0.393	^**^
*rpoD*	1.22	2.33 ± 0.300	^**^
*glnS*	0.94	1.92 ± 0.331	^*^

**Notes.**

* For *p* < 0.5.

** For *p* < 0.01.

*** For *p* < 0.001.

a Genes *dnaK*, *dnaJ* and *grpE* are predicted to be in the same operon, sharing the same *σ*^32^ binding site upstream of *grpE*.

b Genes *groEL* and *groES* are predicted to be in the same operon, sharing the same *σ*^32^ binding site upstream of *groES*.

To identify possible function and efficiency of σ^32^ binding sites, MED whiteflies were treated at two temperatures, 40 °C and 25 °C (as control) for 3 h, and collected immediately. qPCR was conducted on *Portiera* 16S rDNA to quantify the density of *Portiera*, and 14 predicted heat-related genes in MED whitefly (using cDNA) to determine the possible differential expression of them between the heat shock treatment samples and control samples. The density of *Portiera* didn’t significantly increase after heat shock (control vs. heat shock treatment, 1.00 ± 0.114 vs. 1.27 ± 0.187, Mean ± Stand Error Mean, *t*-test, *p* = 0.259). However, all the 15 genes (including *rpoH* encoding σ^32^factor) tested present significant variations (Table 2).

Our results of *Portiera* are quite similar to that of *Buchnera* (Wilcox et al., 2003), that only modest dynamics of gene expression are observed compared to free-living *E. coli*. This is probably due to the weakness of the σ^32^ promoters (Wilcox et al., 2003). On the other hand, we focused on six genes *dnaK*, *groEL*, *groES*, *ftsH*, *dnaJ* and *hslV*, with log2FoldChange >2. The results suggested that genes with predicted σ^32^ binding sites showed higher upregulation under heat shock. One σ^32^ binding site was proposed in the promoter of *grpE*, *dnaK* and *dnaJ.* These three genes were conjunct to each other, and supposed to be in the same operon according to genomic data. This σ^32^ binding site containing promoter may control the transcription of the three genes. Meanwhile, genes *groEL* and *groES* may also be in the same operon and be regulated together, with the σ^32^ binding sites in the upstream of *groES*. However, *ftsH* and *hslV* did not have recognized σ^32^ binding sites in immediate upstream spacer regions. How the transcription of these two genes were induced remains unclear.

Furthermore, we investigated the σ^32^ binding sites in another three available *Portiera* genomes, which are from different whitefly species (Fig. 3). The σ^32^ binding site upstream of *grpE* in 7 *Portiera* genomes showed weak similarities, while σ^32^ binding site close to *groES* presented an almost identical sequence. It is indicated that the regulation of *groES* and *groEL* might be universally kept in *Portiera* of whitefly (including whitefly *Bemisia tabaci*), and thermal tolerance ability may conserved among *Portiera* in different species of whiteflies.

### Putative functional elements in intergenic spacers of *Portiera* genomes

**Figure 4:**
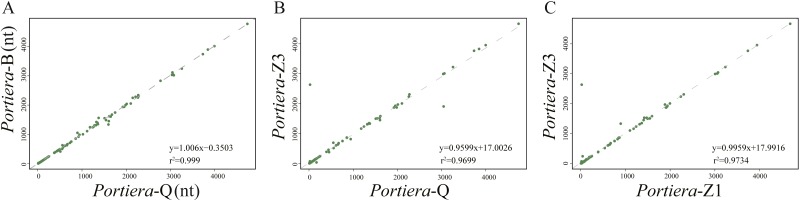
Length associations of orthologous IGSs between different pairs of *Portiera* genomes. (A) Comparison between Portiera-B and Portiera-Q; (B) comparison between Portiera-Z3 and Portiera-Q; and (C) comparison between Portiera-Z3 and Portiera-Z1.

For intergenic spacers, we focused on *Portiera* genomes from whitefly *B. tabaci* species only. Intergenic spacers occupy over 30% of the *Portiera* genomes from whitefly *B. tabaci*, thus leading to the investigation for possible regulatory elements in intergenic spacers. Strong associations between lengths of orthologous IGSs were observed between four *Portiera* genomes (Fig. 4), which suggests that four *Portiera* genomes are coherently arranged even in the non-coding region with only subtle variations, indicating genome stasis (Tamas et al., 2002). Notably, there is a closer relationship between the two invasive species and between the two indigenous species, as evidenced by *r*^2^ values (Fig. 4), which is in agreement with phylogeny (Fig. 1).

Those determined IGSs were then aligned among four *Portiera* from different whitefly species, which identified 95 IGSs (Data S1) orthologous (over 20 nucleotides in length) ranging from 20 to approximately 4700 nucleotides. The conserved loci are quite AT-rich with GC contents only around 23.5%. Furthermore, identically aligned loci among four *Portiera* occupied only a total of 62,438 nt, about 52% of all the orthologous IGSs. The stretch of identically aligned loci was probably interrupted by decayed pseudogenes at varying stages or random mutations in some preserved functional elements. Notably, 51 loci (Data S1) were over 20 nucleotides in length and were subjected to further analysis. In all, 37 perfectly conserved or nearly perfectly conserved Shine-Dalgarno sequences (Data S1) were detected (consensus sequence AGGAG), as well as 34 putative small RNAs predicted (Data S1) by using RNAFold. Since functional elements tend to evolve slower than nonfunctional elements (Ganley & Kobayashi, 2007), the predicted functional loci within IGSs may be involved in the process of transcriptional regulation. The predicted loci need further study for a better understanding of transcriptional regulation within the non-coding region.

Meanwhile, these predicted transcriptional elements were searched against another three *Portiera* genomes available, *Portiera*-TV, and *Portiera* genomes from *A. dispersus* and *A. floccissimus*. Among the 37 Shine-Dalgarno sequences detected in *Portiera* genomes of whitefly, eight of them were also detected in the orthologous IGSs from the additional three *Portiera* genomes, with another three Shine-Dalgarno sequences detected only in one or two additional *Portiera* genomes. Moreover, according to the 34 orthologous IGSs containing putative small RNAs in *Portiera* genomes of whitefly *B. tabaci*, corresponding regions in the extra three *Portiera* genomes were extracted, resulting in 22 orthologous IGS groups (Data S1). Each group contains at least one orthologous IGSs sequence from another three *Portiera* genomes. Interestingly, there were 11 groups, in which seven sequences were predicted to contain a putative small RNA, meaning these sRNAs were universally kept by *Portiera* genomes investigated, and within the orthologous IGS regions. Previous prediction on the divergence of whitefly species, wherein the separation between *Portiera* strains from *A. dispersus* and *A. floccissimus*, and *T. vaporariorum* and *B. tabaci*, was around 129 Ma, implies that the conserved elements found here might have been conserved at least 129 million years. However, the functions of these sRNAs need further investigation.

## Conclusions

Taken as a whole, our study indicates similar function of *Portiera* in whitefly *B. tabaci* and the first to investigate putative transcriptional regulation processes in *Portiera* of whitefly *B. tabaci*. Several regulatory elements in both coding and non-coding regions are revealed, by the combination of orthologous and phylogenetic footprinting approaches. For the coding regions, four *Portiera* genomes retained a similar set of genes that are responsible for transcriptional regulation. In particular, the possible thermal tolerance ability of *Portiera* was revealed genetically and experimentally. Although non-coding regions contain decayed pseudogenes, *Portiera* genomes of whitefly *B. tabaci* still retain functional elements including putative sRNAs. This finding demonstrates regulation within symbiont genomes and gives insights into symbiosis mechanisms.

##  Supplemental Information

10.7717/peerj.7477/supp-1 Table S1 Primers utilized in quantitative PCR

10.7717/peerj.7477/supp-2 Table S2 Sequence similarities (%) between different *Portiera* species pairs

10.7717/peerj.7477/supp-3 Table S3 No retention of regulatory genes for essential amino acid biosynthesis pathways in *Portiera* inferred from *Escherichia coli*

10.7717/peerj.7477/supp-4 Table S4 Calculation of *Ka/Ks* ratio of genes *cspA* between orthologous pairs from different *Portiera* species

10.7717/peerj.7477/supp-5 Data S1 List of 95 intergenic spacers (IGS) orthologs, 51 perfectly aligned IGS (over 20 nucleotides), 37 Shine-Dalgarno sequences and 34 putative small RNAs

## Additional Information and Declarations

### Data Availability

The following information was supplied regarding data availability: The sequences of Portiera from whitefly B. tabaci Asia II 3 (previously referred to as ZHJ1 ‘biotype’) (Portiera BT-Z1) and Portiera from whitefly *B. tabaci* China 1 (previously referred to as ZHJ3 ‘biotype’) (Portiera BT-Z3) are available in GenBank under accession numbers CP016327 and CP016343.
