# Supplementary material for: Conservation of transcriptional elements in the obligate symbiont of the whitefly Bemisia tabaci
Source: PeerJ. 2019 Aug 16;7:e7477. doi: 10.7717/peerj.7477 (PMC6699477; doi:10.7717/peerj.7477)
Supplement: Table S1 [file peerj-07-7477-s001.pdf]

Table S1 Primers utilized in qPCR.

Target gene	Primers
Portiera 16S rRNA	qPor-F: GTGGGGAATAACGTACGG
	qPor-R: CTCAGTCCCAGTGTGGCTG
RPL29 of whitefly	QRPL29-F: TCGGAAAATTACCGTGAG
	QRPL29-R: GAACTTGTGATCTACTCCTCTCG[illegible]
Portiera -*groES*	PgroES-F: ATCGTGTTGTGGTTCGTTGT
	PgroES-R: TCTAAAGGACGGATAGCACCA
Portiera -*dnaK*	PdnaK-F: TCAGAAGGTGCCCGTACTAC
	PdnaK-R: ACTTGTACCCATGCATCTCCA
Portiera -*ftsH*	PftsH-F: GCTTTGTTAAGGCCCGGAAGG
	PftsH-R: CTTTACTTTCCGTTTGGCGGCTA
Portiera -*dnaJ*	PdnaJ-F: TGTGGTGTAAATGGAGGCCC
	PdnaJ-R: CTGTTTGTGTTTCCGCTGGA
Portiera -*grpE*	PgrpE-F: ACCTGTTATTGATGGGCTAGAAA
	PgrpE-R: TGCTTCGTGACATAGTGGATT
Portiera -*hslV*	PhslV-F: GGAGATGGCCAAGTTACACT
	PhslV-R: CGACTGCCAACATAGCTTCT
Portiera -*lon*	Plon-F: ACCTCGCCAGCATTAAAACA
	Plon-R: GCGTCCATGCTAATCCTGTT
Portiera -*ybeY*	PybeY-F: ACTAAGTTCTTGGTTTTGTTCGTT
	PybeY-R: AATTCTTTGCGAAAGAGCTTCA
Portiera -*groEL*	PgroEL-F: GGTGTTGTAGCTGGAGGAGG
	PgroEL-R: GATGCTTCTTCACCTGCGTT
Portiera -*ileS*	PileS-F: TTTGATTCCGGAACAACGCA
	PileS-R: ACGGTGCATGGCCAGATATA
Portiera -*rpoH*	PrpoH-F: CCTGCTCATGGTGTTCGTTT
	PrpoH-R: TCTCTCTTACTACCGTGGGC
Portiera -*valS*	PvalS-F: CGTGTGGAGATCGATCATGTG
	PvalS-R: GTCCCCACCATAACTGCCTA
Portiera -*hslU*	PhslU-F: ACCATCATCTGCGTTAACCAG
	PhslU-R: TGTAATCTACGGGCGCCAAT
Portiera -*rpoD*	PrpoD-F: CGCAGCAACAGATGAAGGAT
	PrpoD-R: TGACGAATTCTTTCACGCGT
Portiera -*glnS*	PglnS-F: GCTAGAAGGCATTACACATTCAA
	PglnS-R: GCCTCTACGTCTCATTCCAGA
