# Supplementary material for: Conservation of transcriptional elements in the obligate symbiont of the whitefly Bemisia tabaci
Source: PeerJ. 2019 Aug 16;7:e7477. doi: 10.7717/peerj.7477 (PMC6699477; doi:10.7717/peerj.7477)
Supplement: Table S2 [file peerj-07-7477-s002.pdf]

Table S2 Sequence similarities (%) between different *Portiera* species pairs.

	***Portiera* -B**	***Portiera* -Q**	***Portiera* -Z1**	***Portiera* -Z3**	***Portiera* -TV**
***Portiera* -B**	100	99.8	98.8	99.1	76.9
***Portiera* -Q**		100	99	99	76.9
***Portiera* -Z1**			100	99.5	77
***Portiera* -Z3**				100	77.8
***Portiera* -TV**					100
