# Supplementary material for: Conservation of transcriptional elements in the obligate symbiont of the whitefly Bemisia tabaci
Source: PeerJ. 2019 Aug 16;7:e7477. doi: 10.7717/peerj.7477 (PMC6699477; doi:10.7717/peerj.7477)
Supplement: Table S3 [file peerj-07-7477-s003.pdf]

Table S3 Retention of regulatory genes for essential amino acid biosynthesis pathways in *Portiera* inferred from *E. coli*

**Regulatory gene**	**Related EAA**	**P/A[a] in *Portiera***
argR	Arg	-
himA	Arg, Val/Ile/Leu	-
lysR	Lys	-
thrL	Thr	-
ilvY	Val/Ile/Leu	-
lrp	Val/Ile/Leu,Trp	-
ilvL	Val/Ile/Leu	-
leuL	Leu	-
leuO	Leu	-
trpR	Trp	-
trpL	Trp	-
pheL	Phe	-
hisL	His	-
metJ	Met	-
metR	Met	-

a. Presence or absence. '-' means absence.
