# Supplementary material for: Conservation of transcriptional elements in the obligate symbiont of the whitefly Bemisia tabaci
Source: PeerJ. 2019 Aug 16;7:e7477. doi: 10.7717/peerj.7477 (PMC6699477; doi:10.7717/peerj.7477)
Supplement: Table S4 [file peerj-07-7477-s004.pdf]

Table S4 Calculation of *Ka/Ks* ratio of gene *cspA* between orthologous pairs from different *Portiera* species.

**Gene pairs**	***Ka***	***Ks***	***Ka/Ks***
Q-TV	0.0347	0.5497	0.0631
B-TV	0.0347	0.5497	0.0631
Z1-TV	0.0251	0.5669	0.0443
Z3-TV	0.0251	0.5669	0.0443
